# Enhanced co-generation of cellulosic ethanol and methane with the starch/sugar-rich waste mixtures and Tween 80 in fed-batch mode

**DOI:** 10.1186/s13068-019-1562-0

**Published:** 2019-09-23

**Authors:** Meishan Fan, Jun Li, Guican Bi, Guangying Ye, Hongdan Zhang, Jun Xie

**Affiliations:** 1 College of Forestry and Landscape Architecture, Guangdong Engineering Technology Research Center of Agricultural and Forestry Biomass, Key Laboratory of Energy Plants Resource and Utilization, Ministry of Agriculture and Rural Affairs, South China Agricultural University, Guangzhou, 510642 China; 2 School of International Relations, Sun Yat-sen University, Guangzhou, China

**Keywords:** Sugarcane bagasse, Cellulose–starch–sugar waste, High solids loading, Fed batch, Bioethanol, Biomethane

## Abstract

**Background:**

The mixed-feedstock fermentation is a promising approach to enhancing the co-generation of cellulosic ethanol and methane from sugarcane bagasse (SCB) and molasses. However, the unmatched supply of the SCB and molasses remains a main obstacle built upon binary feedstock. Here, we propose a cellulose–starch–sugar ternary waste combinatory approach to overcome this bottleneck by integrating the starch-rich waste of *Dioscorea composita* Hemls. extracted residue (DER) in mixed fermentation.

**Results:**

The substrates of the pretreated SCB, DER and molasses with varying ratios were conducted at a relatively low solids loading of 12%, and the optimal mixture ratio of 1:0.5:0.5 for the pretreated SCB/DER/molasses was determined by evaluating the ethanol concentration and yield. Nevertheless, it was found that the ethanol yield decreased from 79.19 ± 0.20 to 62.31 ± 0.61% when the solids loading increased from 12 to 44% in batch modes, regardless of the fact that the co-fermentation of three-component feedstock was performed under the optimal condition defined above. Hence, different fermentation processes such as fed-batch and fed-batch + Tween 80 were implemented to further improve the ethanol concentration and yield at higher solids loading ranging between 36 and 44%. The highest ethanol concentration of 91.82 ± 0.86 g/L (69.33 ± 0.46% of theoretical yield) was obtained with fed-batch + Tween 80 mode during the simultaneous saccharification and fermentation at a high solids loading of 44%. Moreover, after the ethanol recovery, the remaining stillage was digested for biomethane production and finally yielded 320.72 ± 6.98 mL/g of volatile solids.

**Conclusions:**

Integrated DER into the combination of SCB and molasses would be beneficial for ethanol production. The co-generation of bioethanol and biomethane by mixed cellulose–starch–sugar waste turns out to be a sustainable solution to improve the overall efficacy in biorefinery.
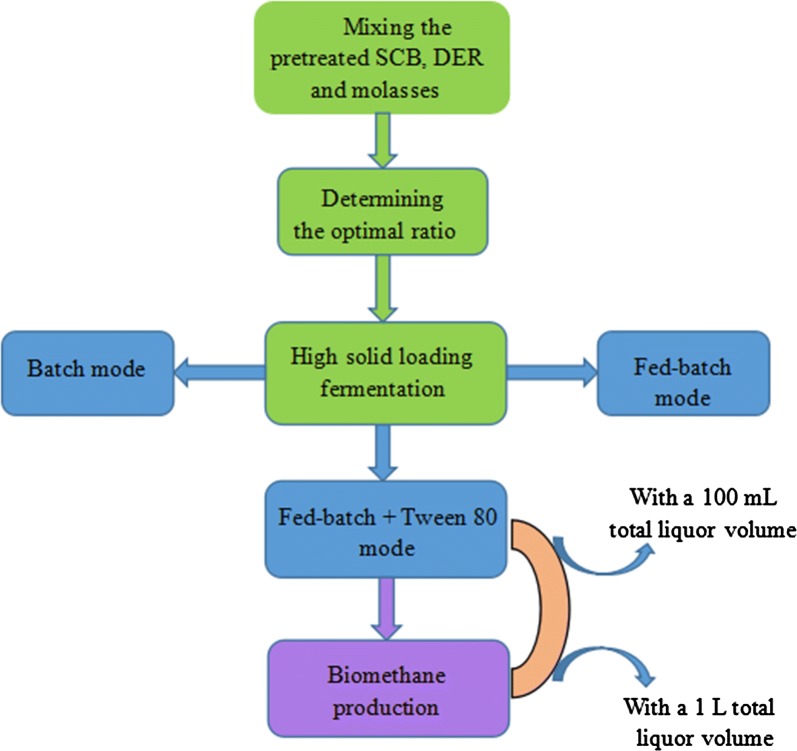

## Background

Today, the world’s transport system is still heavily dependent upon fossil fuels supply despite the rapid growth of alternative technologies such as electric vehicles (EVs), compressed natural gas (CNG) cars or biofuels including both bioethanol and biodiesel^1^ [1]. The use of fossil fuels has brought about a series of problems, such as environment concerns, depletion of conventional crude oil reserves and regional conflicts of resource control [2, 3]. Biofuels, as alternative energy resources, can relieve those constraints, of which bioethanol plays an important role and accounts for more than 90% of total biofuel utilization [4, 5]. IPCC (2018) has clearly indicated the necessity of transitioning world energy system rapidly to the negative emissions pathways to achieve the global 1.5–2 °C target by the middle of the twenty-first century^2^ [6], wherein biofuels are destined to play an increasingly important role in decarbonizing the transport sector over the next decades [7]. This is particularly relevant in many developing countries such as China who are faced with a double challenge of ensuring food and energy security. Currently, nearly 70% of Chinese oil consumption depends on imports and over half of them goes to the transport sector in 2017 [1, 8]. There is thus an urgent need to accelerate domestic noncrop resources-based bioethanol and biofuel production technologies in the nation.

China has introduced a series of biofuel targets and relevant policy support since 2001, such as the production of bioethanol and utilization of E10 automobile fuel, and has become the third largest producer and consumer of bioethanol, just behind the USA and Brazil [9, 10]. However, bioethanol production based on food crops is being widely criticized since it causes the diversion of edible crops and the increase in food prices [11, 12]. The development of noncrop-based feedstock for bioethanol production has been considered an urgent policy agenda by the National Development and Reform Commission (NDRC), the main body of China’s energy policy making [13]. Currently, lignocellulosic bioethanol production has aroused great research interest as the feedstocks are cheap and abundant while avoiding the competition with food sources [14, 15]. However, the commercial production of bioethanol still remains at a nascent stage due to high enzyme costs and low efficacy of generation (both titers and yields) [16].

More recently, the concept of mixed feedstocks, i.e., combining two or more different substrates, has been proposed and investigated to improve the production of lignocellulosic ethanol [3]. The selection of substrates should be based on the requirement of avoiding extra nutrient supplementation, the proximity of the different feedstocks to the collection center or processing facility, and the overall economic viability in term of the relative abundance and low cost of feedstock [3, 17]. Moreover, the selection of mixture components in the current literature mainly referred to the combinations of diverse lignocellulosic biomass or the integration of sugar and or starch-based component into lignocellulosic feedstock [18]. The mixture of different lignocellulosic materials has been researched by combining the substrates classified either as the same category (e.g., mixed hardwood [19], grasses mixtures [20]) or different category (sugarcane bagasse + sugarcane straw [21], rice straw + wheat bran [22]). Meanwhile, the integration of sugar or starch-based component into lignocellulosic ethanol production has also been investigated, wherein the mix component included first-generation biomass feedstock (e.g., wheat meal [23], corn kernel [24]) and starch/sugar-rich waste (e.g., *Dioscorea composita* Hemls. extracted residue (DER) [25], molasses [26]). This process allows us to reduce the production cost and to increase the lignocellulosic ethanol generation [27].

Our previous work [26] integrated molasses into SCB-based ethanol production which enhanced final ethanol generation and demonstrated that the optimal ratio of sugarcane bagasse and molasses for fermentation was 1:1. Nevertheless, the annual productions of molasses and sugarcane bagasse (SCB) in China’s sugar industry is 4 million and 36 million tons, respectively [28]. This output ratio discrepancy makes molasses incapable of meeting the requirement of SCB-based bioethanol production. To overcome this feedstock imbalance issue, cassava and DER are considered to replace a part of the molasses because they are both starch rich and cultivated worldwide in subtropical and tropical regions. However, cassava, as one of the main materials for starch production in industry, is also consumed as staple food in some regions such as South Asia and Latin America [29], which therefore rules out its large application for biofuel production as feedstock.

So, this paper attempts to integrate DER, a starch-rich supplement, into the combination of SCB and molasses for ethanol production, as *D. composita* Hemls., a robust energy and medicine plant adapted to local climate and soils in southern China, can be grown at large scale and processed with sugarcane in the same region, and its abundant DER can be easily harvested with SCB at the same time [25]. The three feedstocks can serve as ideal substrates for bioethanol generation due to their advantages of guaranteed availability (they are abundant carbohydrates which can be produced and collected at the same place) and accessibility at low cost (industry waste).

As an abundant and renewable lignocellulosic biomass, SCB is mainly composed of cellulose, hemicellulose, and lignin; the latter has been identified as the major factor of inhibiting cellulase hydrolysis, due to its irreversible and non-productive adsorption to cellulase [30]. To effectively remove the recalcitrant component, alkali pretreatment was carried out to disrupt the ether and ester bonds in the lignin units and break the linkages among lignin, cellulose and hemicellulose [31].

The simultaneous saccharification and fermentation (SSF) have been widely used to reduce the operational process and cost. In addition, high solids loading fermentation was employed to gain higher ethanol concentration (≥ 40 g/L) and meet the economic production requirement [32]. However, some technical challenges were also brought about with high biomass loading, such as the difficulties of stirring and mixing, the limitation of mass transmission, and the prolonged fermentation time [33]. Fed-batch mode has been proved to hold a good potential for solving those difficulties and improving the ethanol production. Moreover, previous literature indicated that supplementing Tween 80 presented positive influence on the enzymatic hydrolysis and SSF fermentation process [33]. Several studies of combining Tween 80 with SSF had been applied to increase ethanol production [25, 34]; however, the comparison of three modes of batch, fed-batch, and fed-batch + Tween 80 at high solids loading with SSF has rarely been reported.

The sequential production of bioethanol and biomethane proposed in this study has been employed as a sustainable approach given that it can generate higher energy yields compared with a single product, while minimizing the environmental impacts caused by the stillage in the ethanol production [35, 36]. Biomethane production by anaerobic digestion is a complex process, which is significantly influenced by substrate characteristics, fermentation condition, and equipment design [37]. Therefore, materials with a high organic content, optimal pH level, and temperature as well as favorable anaerobic environment were necessary to meet the nutritional requirement of the microbes during the entire production process. Also, the high content of organic matter in the distilled waste feedstocks generated during the ethanol production can be successfully degraded by bacteria, which is conducive to the increase in bioethanol output. In most of the previous studies, the remaining residues following the ethanol recovery were applied to anaerobic digestion to generate environmental benefits such as a significant reduction of COD (around 70–95%).

In this article, DER is used to replace a part of molasses to satisfy the molasses/SCB ratio requirement for ethanol production. This new framework of noncrop plant and biomass waste-based feedstock mixture is then applied to ethanol production and methane recuperation. The optimal designs are characterized by ternary mixed feedstock combinations of DER, SCB, and molasses. After evaluating the optimal ratio of dilute alkali-pretreated SCB, molasses, and DER at a relatively low solids loading of 12%, the co-fermentation of ternary mixed feedstocks with the optimal ratio was performed at solids loading from 12 to 44% in batch mode. Furthermore, batch, fed-batch, and fed-batch + Tween 80 with SSF were performed to assess a more efficient ethanol conversion at higher solids loading (> 32%). Specifically, the stillage was subsequently digested for biomethane production as an effective way to improving the overall efficiency of the biorefinery.

## Results and discussion

### Chemical analysis of materials

As shown in Table 1, molasses, as the sugar-rich residue of sugar processing, contained 9.02 ± 0.40% fructose, 6.04 ± 0.20% glucose, and 23.04 ± 0.70% sucrose (w/w). DER used in this study is mainly composed of 9.81 ± 0.21% of cellulose, 44.70 ± 0.56% of starch, 15.10 ± 0.11% of xylan, and 11.02 ± 0.52% of acid-insoluble lignin (AIL). The cellulose in pre-saccharified DER was also hydrolyzed to produce ethanol due to the existing cellulase in the co-fermentation condition. The raw SCB mainly consisted of 37.93 ± 0.43% cellulose, 23.25 ± 0.33% xylan, 26.03 ± 0.21% AIL, and 6.34 ± 0.31% acid-soluble lignin (ASL). After dilute alkali pretreatment, the lignin (including AIL and ASL) content in the remaining solids decreased to 10.05 ± 0.05% and 4.01 ± 0.03%, whereas the contents of cellulose and xylan in pretreated SCB increased to 53.01 ± 0.70% and 29.36 ± 0.30%, respectively. The delignification during dilute alkali pretreatment would break down the intact structure of raw SCB, and the decreased content of lignin would reduce the enzymolysis barrier caused by lignin. Meanwhile, these changes would make cellulose partially exposed to enzyme and present a positive effect on the enzymatic hydrolysis and fermentation [38].

**Table 1:** The chemical composition of the substrates (%)

Materials	Cellulose^a^	Starch^a^	Xylan^a^	AIL^a^	ASL^a^	Fructose^b^	Glucose^b^	Sucrose^b^	Solid yield
Molasses						9.02 ± 0.40	6.04 ± 0.20	23.04 ± 0.70	30.61 ± 0.10^b^
DER	9.81 ± 0.21	44.70 ± 0.56	15.10 ± 0.11	11.02 ± 0.52					
Raw SCB	37.93 ± 0.43		23.25 ± 0.33	26.03 ± 0.21	6.34 ± 0.31				93.26 ± 0.10^a^
Pretreated SCB	53.01 ± 0.70		29.36 ± 0.30	10.05 ± 0.05	4.01 ± 0.03				81.60 ± 0.30^a^

^a^Refers to dry basis

^b^Refers to wet basis

*AIL* acid-insoluble lignin, *ASL* acid-soluble lignin

### The experiment of optimizing mix ratios of feedstocks on SSF

**Fig. 1:**
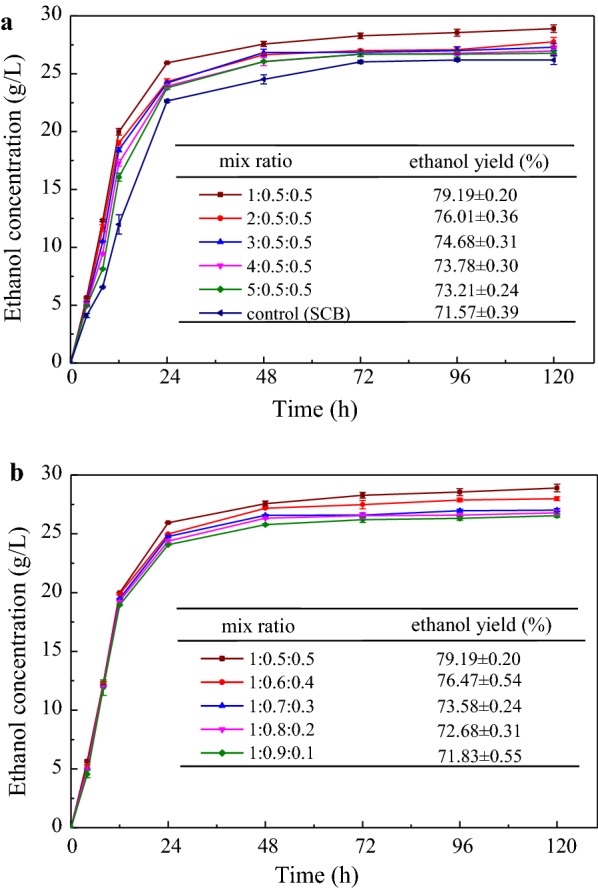
The ethanol concentration/yield during SSF of the pretreated SCB, DER, and molasses at 12% solids loading

To overcome the ratio imbalance of molasses and SCB output during sugar processing, this study integrated DER into the ethanol production of SCB and molasses. Determining the appropriate materials ratio is necessary to make it better applied into the process of high solids fermentation. Firstly, to increase the input of SCB, the ratios of pretreated SCB/DER/molasses were set at 1:0.5:0.5, 2:0.5:0.5, 3:0.5:0.5, 4:0.5:0.5, and 5:0.5:0.5 (dry weight) with a solid loading of 12%. Figure 1 shows the ethanol concentration at different fermentation times and ethanol yield at the end of fermentation (120 h). The change of all ethanol concentrations has a similar trend that sharply increased in the first 24 h then rose gradually to steady until 120 h. Because the fermentable sugar obtained from molasses and pre-saccharified DER in the slurry can be directly fermented by the yeast, significant differences of fermentation rates were found in the initial 12 h between the control and the mixtures. Compared to single SCB fermentation, the addition of DER and molasses can accelerate the rate of ethanol formation, especially in the first 12 h. High fermentation rates for the mixtures during the first 12 h (1.33–1.66 g/L/h) were observed, whereas the control of pure pretreated SCB showed slow ethanol productivity of only 0.99 g/L/h at the same time. The ethanol production became slow after 24 h and the final ethanol concentrations from 28.89 ± 0.32 to 26.76 ± 0.24 g/L were achieved for the ratio of 1:0.5:0.5, 2:0.5:0.5, 3:0.5:0.5, 4:0.5:0.5, and 5:0.5:0.5 (pretreated SCB/DER/molasses), respectively. Meanwhile, fermentation concentration of 26.19 ± 0.39 g/L could be obtained from pure SCB. The highest ethanol concentration (28.89 ± 0.32 g/L) and yield (79.19 ± 0.20%) were obtained with the ratio of 1:0.5:0.5 for pretreated SCB/DER/molasses. This may be explained by the fact that starch was easier to be hydrolyzed than cellulose, and a greater amount of molasses and pre-saccharified DER provided more favorable nutrients for the yeast growth and fermentation [39]. Moreover, further experiment should be conducted to optimize the appropriate proportion of DER and molasses.

Based on the ratio of 1:0.5:0.5 for pretreated SCB/DER/molasses, a greater amount of pre-saccharified DER was used to replace molasses with the ratio of 1:0.5:0.5, 1:0.6:0.4, 1:0.7:0.3, 1:0.8:0.2, 1:0.9:0.1. As shown in Fig. 1b, the fermentation rate increased with the greater amounts of molasses, because the fermentation of fermentable sugar in molasses is usually faster and can be completed within 24 h. However, the saccharification of starch and cellulosic hydrolysis from DER requires longer time. Although significant differences could not be found among the ethanol concentrations, the ethanol yield was significantly increased when a greater amount of molasses was added [26]. These results were attributed to the higher sugar content from the starch and cellulose in DER. As a result, the optimal ratios of 1:0.5:0.5 for the pretreated SCB/DER/molasses were determined based on the highest ethanol concentration and yield.

### The experiment of increasing ethanol concentration at high solids loading

**Fig. 2:**
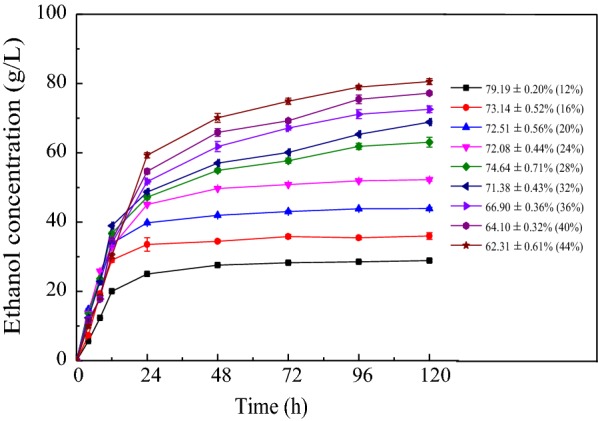
The ethanol concentration/yield during SSF, with solids loading (W/W) appearing in parentheses

Fermentation with high solids loading was an effective way to increase ethanol concentration [40], hence, in this section, different substrates loading of 12–44% with the optimal ratio of 1:0.5:0.5 for the pretreated SCB/DER/molasses were investigated to explore their influence on SSF. Figure 2 depicts the time course of ethanol concentration and a final ethanol yield after 120 h during SSF. As shown, solids loading of substrates presented an obvious influence on ethanol productivity. It was observed that all ethanol titers increased sharply in the first 24 h and then slowly rose to the highest value. At the low solids loading from 12 to 24%, the ethanol concentration exhibited insignificant variations after 24 h. However, when solids loading were increased from 28 to 44%, it needed more time (120 h) to produce ethanol because high sugar content would take more time for yeast to convert into ethanol [25]. Furthermore, the fermentation rate increased as the increment of solid loading at 12 h reached the highest rate of 3.25 g/L/h with 32% solids loading. However, when the solid loading exceeded 32%, the fermentation rate started to decline instead of increasing further. It can be concluded that the limited system liquidity at a high solid loading led to inadequate stir and ineffective hydrolysis and fermentation at the initial time. Similar findings have been reported in previous literature [25, 41].

The final ethanol concentrations with 12–44% solids loading was 28.89 ± 0.32 g/L, 35.98 ± 0.96 g/L, 43.86 ± 0.39 g/L, 52.24 ± 0.44 g/L, 63.04 ± 1.40 g/L, 68.84 ± 0.21 g/L, 72.55 ± 0.96 g/L, 77.20 ± 0.40 g/L, and 80.56 ± 0.84 g/L, respectively. When the solid loading was over 16%, the ethanol concentrations were higher than 40 g/L, reaching the economical distillation titers. However, the ethanol yield decreased from 79.19 ± 0.20 to 62.31 ± 0.61% for 12–44% solids loading, indicating that many substrates could not be utilized during high solids fermentation. A low ethanol yield with high solids loading could be explained by the low hydrolytic efficiency of cellulase due to non-productive adsorption on lignin and decreased sensitivity of yeast because of the accumulation of inhibitors at high solids loading [42]. Although ethanol concentration significantly increased with an increasing solid loading, the final ethanol yield decreased when more substrates were used into fermentation in batch mode, which made the process costs increase and the economic efficiency reduce [43]. Hence, it was necessary to discuss the influence of different fermentation experiments (fed-batch and fed-batch + Tween 80) on ethanol concentration and yield.

### The experiment of improving ethanol concentration and yield at high solids loading

**Fig. 3:**
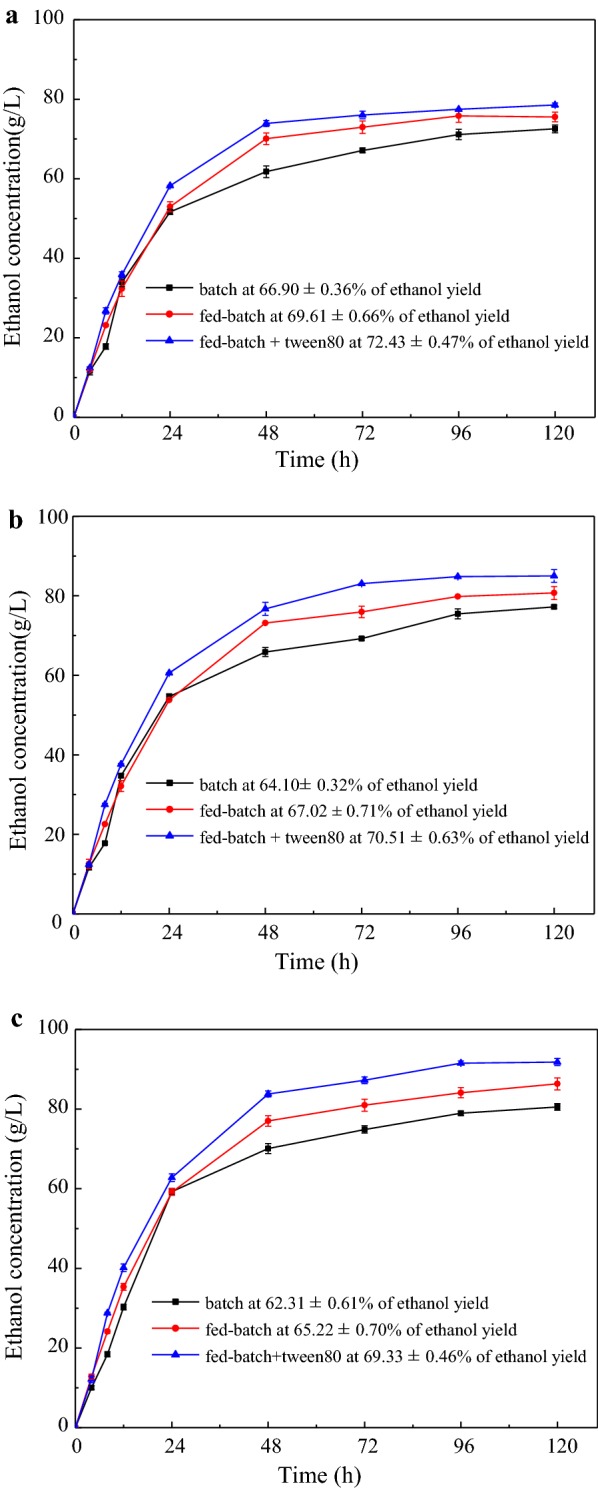
Comparison of the ethanol concentration/yield in “batch”, “fed-batch”, and “fed-batch + Tween80″ modes during SSF at solids loading (w/w) of 36% (**a**), 40% (**b**), and 44% (**c**)

Fed-batch mode is considered as a favorable way to increase cell contents and facilitate the ethanol concentration accumulation [44, 45]. The fed-batch and fed-batch + Tween 80 have been chosen as an instrument to conduct the following experiments of gradually feeding biomass into the fermentation tank to reduce the above-mentioned negative effect of batch mode at high solids loading. During fed-batch SSF, the feeding mode of substrates, yeast, and enzymes have great effect on the entire reaction process. Liu et al. found that all the addition of yeast at the beginning of SSF achieved higher ethanol productivity [46]. Gao et al. evaluated that all cellulase added at 0 h was more favorable to the fermentation process [47]. In this study, all enzymes and yeast were added at the start of SSF and the optimal substrates feeding methods are shown in Table 2. Because pre-saccharified DER required a certain amount of water, partial SCB and all DER were added at 0 h to make a minimum initial solid loading. With the initial solids loading of 21.2%, 22.9%, and 24% for the solids loading of 36% (Fig. 3a), 40% (Fig. 3b) and 44% (Fig. 3c), respectively, the fermentation rates were higher than that of batch mode at the first 8 h due to the increment of system liquidity and exposing more available catalytic sites [48].

**Table 2:** The processes of substrates feeding

	SCB	DER	Molasses
(i)			
36%	18	9	9
40%	20	10	10
44%	22	11	11

	36%	40%	44%
(ii)			
T_0_	6^a^ + 9^b^	6^a^ + 10^b^	6^a^ + 11^b^
T_12_	6^a^	8^a^	10^a^
T_24_	6^a^ + 9^c^	6^a^ + 10^c^	6^a^ + 11^c^
Initial solids loading	21.2%	22.9%	24%

(i) The final substrates mixture in term of gram number of pretreated SCB/DER/molasses in final 100 mL of total fermentation water. (ii) The feed amount of substrates at different fermentation times (T_h_)

^a^SCB

^b^DER

^c^Molasses

However, the ethanol concentration did not show significant differences between the batch mode and fed-batch mode with solids loading of 36%, 40% and 44% at 24 h, it may be explained that the substrates fed has not be completed and the fermentation system have not reached the final solids loading before collected samples were determined at 24 h. There was a significant increase of fed-batch mode compared with batch mode after 24 h, because the addition of molasses at 24 h showed superior fermentation efficiency in fed-batch mode and insufficient mixing and deficient enzymatic accessibility sites in batch mode. Thereafter, the ethanol titers increased gradually until 120 h and the final ethanol concentrations were 72.55 ± 0.96 g/L (75.55 ± 1.22 g/L), 77.20 ± 0.40 g/L (80.69 ± 1.62 g/L), and 80.56 ± 0.84 g/L (86.33 ± 1.49 g/L) for the batch (fed-batch) mode at the solids loading of 36%, 40% and 44%, respectively. The ethanol yields were improved from 66.90 ± 0.36 to 69.61 ± 0.66% (36% of solids loading), 64.10 ± 0.32% to 67.02 ± 0.71% (40% of solids loading), and 62.31 ± 0.61% to 65.22 ± 0.70% (44% of solids loading), respectively. During the fed-batch process, the solid loading was always kept in low condition, leading to the high ratio of cellulase and yeast to substrates, which improved the ethanol concentration and yield.

Tween 80 as a kind of surfactant was added to the fed-batch SSF to further improve the fermentation efficiency [49]. The feeding method of fed-batch + Tween 80 was the same as that for the mode of fed-batch except for the addition of Tween 80 (100 mg/g substance) at the beginning of the experiment, and the fermentation results are shown in Fig. 3. Their fermentation rates were higher than those of the fed-batch and batch mode in the total fermentation process; this phenomenon can be explained intwo ways: the first was Tween 80 could reduce the surface tension of the liquid and thus increase the reactive contact between substances; the second was the combination of Tween 80 and lignin could reduce the unproductive absorption of lignin on cellulase [49]. The final high ethanol concentrations of 80.56 ± 0.84 g/L, 86.33 ± 1.49 g/L and 91.82 ± 0.86 g/L were achieved at the loading of 36%, 40% and 44%, respectively. Although the ethanol concentration increased gradually from 36 to 44%, the ethanol yield decreased from 72.43 ± 0.47 to 69.33 ± 0.46%. This is likely to be the accumulation of by-production and the non-productive adsorption at a higher solids loading [26, 42]. Compared to the batch mode, the fed-batch + Tween 80 mode could generate the highest ethanol concentration of 91.82 ± 0.86 g/L with conversion yield of 69.33 ± 0.46%. It could be concluded that fed-batch + Tween 80 was an efficient way to improve the ethanol productivity at high solids loading fermentation process.

### The experiment of anaerobic digestion of the residual stillage

**Fig. 4:**
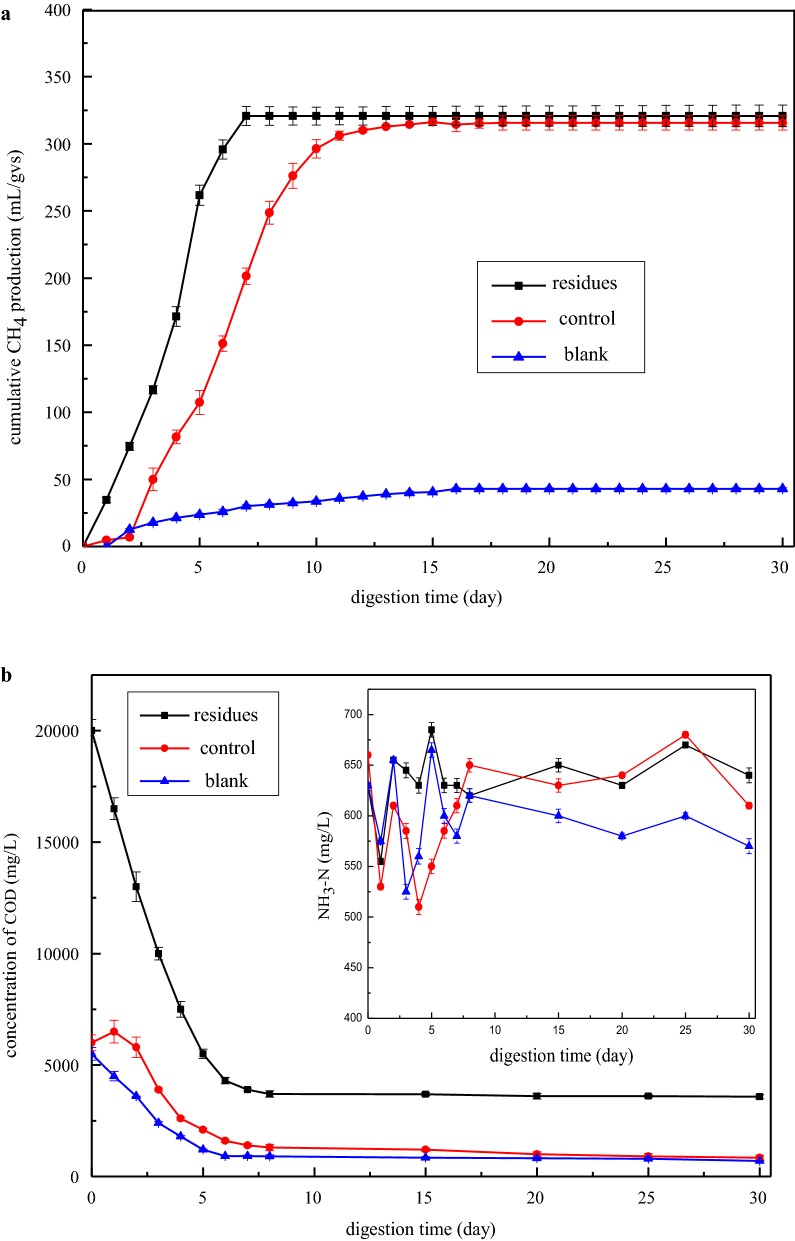
The variations of cumulative methane production (**a**), COD, and NH_3_-N value (**b**) during the anaerobic digestion process

The experiment of biomethane production was continuously conducted after recovering ethanol by evaporation in fed-batch + Tween 80 mode at 44% solids loading. Previous literature reported that the residual sugars and some fermentation by-products were popular for the inoculum to convert methane [36, 50]. As shown in Fig. 4a, the inoculum showed a good methanogenic activity which was deduced from the positive control experiment using microcrystalline cellulose as the substrate with BMP value of 315.65 ± 5.35 mL/g VS. It was observed that the maximum methane yield of 320.72 ± 6.98 mL/g VS was produced after 30 days of digestion from the residual stillage. Similarly, our previous research evaluated the sequential biofuel production by integrating molasses into sugarcane bagasse and obtaining 312.14 mL/g VS methane. A higher methane productivity was achieved in this study because of the three ternary mixture characteristics and relatively low ethanol fermentation efficiency, which left more residual organic contents. In addition, in the fermentation process, the NH_3_-N values of experimental, control, and blank groups were steady, and they were between the range of (500 and 700) mg/L, which will not inhibit the production of methane in Fig. 4b [51]. The final COD removal efficiency of 82.14 ± 0.75% with an initial concentration of 20,000 mg/L and VS degradation yield of 94.30 ± 0.63% further suggested that most of the fermentation residuals could be converted to biomethane.

It was reported that every liter ethanol produced would generate 7.8 L of stillage [50]. These vinasse can be treated by incineration or conversion into animal feed [52]. However, this method of incineration would generate more cost and energy consumption due to the high content of moisture. Conversion into animal feed cannot match the required protein and fiber content. Therefore, the anaerobic digestion of stillage was developed as an efficient and environmentally friendly method. The combined experiments of bioethanol and biomethane were also performed in a final 1 L of total liquor volumes at 44% of total solids loading to analyze the feasibility of cellulose to glucose to ethanol conversion, apparent viscosity, and the production of biofuel thermal value. For the 1 L liquor volume experiment, the thermal value obtained from the generated ethanol of 90.83 g was 2724.90 kJ, and the produced 37.19 L methane released 1335.12 kJ based on the heat values of ethanol (30.0 kJ/g) and methane (35.9 kJ/L) [36]. Comparative research of biofuel (bioethanol and methane) co-production from different substrates based on high solids fermentation has been summarized in Table 3. Overall, the results obtained in this study lie in the same range as those found in other studies. It was observed that higher energy values were achieved in the co-production process than ethanol fermentation alone. This indicated that sequential biofuel co-production would be a more favorable alternative for comprehensive utilization of biomass.

**Table 3:** Comparison of biofuel co-production (bioethanol and methane) yields from different substrates based on high solids fermentation

Material	Bioethanol production			Methane production		References
	Solid loading	Ethanol concentration (g/L)	Ethanol yield (%)	VS ratio (inoculum:substrate)	Methane yield (mL/g vs)	
Birch wood	35% (w/w)	83.2	68.7	1:1	188.1	[36]
Corn stover	24% (wt%)	76	78.3	2:1	120	[57]
Corn stover	35.5% (w/w)	70.7	72.5	No description	320	[58]
SCB	30% (w/v)	68.047	74.13	1:1	306.974	[50]
SCB + molasses	36% (w/v)	94.20	72.37	1:1	312.14	[26]
DER + SCB + molasses	44% (w/w)	91.82	69.33	1:1	320.72	This study

## Conclusions

This article used the starch-rich waste of *D. composita* Hemls. to substitute a part of molasses to improve SCB-based ethanol production. The ternary combination of cellulose–starch–sugar waste allows overcoming the unmatched supply of molasses and SCB output during sugar processing. At the optimal ratio of 1:0.5:0.5 for the pretreated SCB/DER/molasses, the solids loading was adjusted from 12 to 44% to increase the final ethanol concentration in batch mode. However, the ethanol yield (in terms of ratio of real output to theoretical yield) declined due to the limited system liquidity under high solids loading. Fed-batch and fed-batch + Tween 80 (100 mg/g substance) were applied to further improve the ethanol concentration and yield at higher solids loading ranging between 36 and 44%. Applying Tween 80 to gradual feeding of substrates allowed the ethanol production efficiency to increase as a result of the moderating effect of yeast sensitivity to high concentrated sugars and system viscosity at high solids loading (> 32%). The maximum ethanol concentration of 91.82 ± 0.86 g/L with a theoretical yield of 69.33 ± 0.46% was obtained after 120 h fermentation under fed-batch + Tween 80 mode. After distillation, the residual stillage was converted to methane by anaerobic digestion, and daily methane production reached a plateau a week after and generated an accumulative yield of 320.72 ± 6.98 mL/g VS for 30 days. In conclusion, the technical roadmap of multi-feedstock biofuels production proposed in the present research has significantly improved the bioethanol generation efficiency by increasing ethanol concentration. It can also overcome the obstacles of low solids loading associated with single raw material and insufficient proportion between SCB and molasses during the co-fermentation process. Furthermore, co-generation of bioethanol and biomethane provides a new perspective of achieving higher energy efficiency and product diversity in industry-scale biomass resources utilization.

## Methods

### Materials

The SCB, molasses, and DER were all obtained from Maoyuan Sugar Co., Ltd., in Shaoguan, Guangdong, China. Pre-milled and screened (< 1 mm lengths) SCB was stored at room temperature, and molasses was kept in a refrigerator at 4 °C. The chemical composition contents of raw and pretreated SCB were analyzed according to the method developed by the National Renewable Energy Laboratory (NREL) [53]. The starch content of DER was determined by a two-step enzymatic hydrolysis method with 20% dry matter after drying at 60 °C for 12 h: liquefaction using amylase enzyme with 150 U/g dry matter (DM) at 85 °C, pH 5.5, for 3 h and saccharification using glucoamylase with 20 U/g DM at 60 °C, pH 4.5, for 24 h [23]. After the enzymatic hydrolysis, the glucose in the supernatant was analyzed using HPLC and the other compositions in the solid residue were determined using the previously mentioned method provided by NREL [53].

Cellulase (Cellic CTec2) with an activity of 164 FPU/mL was used for SSF according to the methods provided by NREL [54].

The *Saccharomyces cerevisiae* used during SSF was provided by Angel Yeast Co., Ltd. (Yichang, China).

### Alkaline pretreatment

Alkaline pretreatment was carried out in a round-bottom flask. The weighted biomass samples were pretreated by 0.5 mol/L NaOH at 80 °C for 2 h with a solid/liquid ratio of 1:20. After pretreatment, the liquor and residues in the slurry were separated by vacuum filtration and the residues were washed until a neutral pH. The pretreated SCB was then subject to composition analysis and SSF after dried at 50 °C for 48 h.

### Pre-saccharification

The weighted DER mixed with deionized water at 250 g/L (w/v) was preheated to 85 °C when the pH was adjusted to 5.5. Meanwhile, 150 U/g DER of liquefying enzyme was added into the DER slurry for 2 h with 120 rpm. Then, when the temperature of mashes was cooled to 60 °C, the saccharification was conducted by using amyloglucosidase (20 U/g DER) for 2 h before the pH of the slurry was adjusted to 4.5.

### Simultaneous saccharification and fermentation (SSF)

During the SSF process, various mixtures of calculated weight of alkali-pretreated SCB, DER, and molasses were added into a 250 mL flask under 4.5 pH (adjusted by 1.0 mol/L of sulfuric acid). Before SSF, the inoculated yeast of 6.6 g was firstly activated at 36 °C for 10 min and 34 °C for 60 min in a 2% glucose solution (100 mL) at 120 rpm. 5 mL of activated yeast and 15 FPU/g pretreated SCB of the cellulase were then injected to the reaction system and finally a specified volume of sterile water was supplied to reach 100 mL of liquor. All experiments were performed in a sterile environment. Different from the batch mode, the fed-batch mode is conducted by gradually feeding the substrates to achieve final high solid loading. The fed-batch + Tween 80 mode is the same as the fed-batch mode except for the addition of Tween 80 (100 mg/g substance) to the system at the initial time. SSF was conducted in the shaker at 34 °C and 120 rpm for 120 h in triplicate.

### Analytical methods

The fermentation sugar content of molasses and saccharified DER was analyzed by a high-performance liquid chromatography system (HPLC, Shimadzu, Japan) equipped with a refractive index detector (RID) and a cation-exchange column (SUGAR KS-801; 300 mm * 8.0 mm; Shodex™, Japan). Ethanol samples were collected periodically and determined by HPLC after centrifugation and purification by 0.22 mm filter. The HPLC uses a refractive index detector (RID) and a cation-exchange column (SUGAR SH1011; 300 mm * 8.0 mm; Shodex™, Japan), and the mobile phase uses 0.05 M H_2_SO_4_ with 1.0 mL/min flow rate at 50 °C. In the SSF, the maximal ethanol yield for glucose (0.51 g/g) could be calculated, and 1.11 g of glucose was produced by 1 g of starch or cellulose. The ethanol yield was calculated by the following formula:

$${\text{Ethanol}}\;{\text{yield}}\,(\% ) = \frac{{{\text{actual}}\;{\text{ethanol}}\;{\text{released}}}}{{{\text{theoretical}}\;{\text{ethanol}}\;{\text{released}}}} \times 100\% .$$

### Biomethane production

After ethanol was evaporated from the fermentation medium using a rotary evaporator at 60 °C, the non-fermented residues were subjected to biomethane production using Bioprocess Control AMPTS II (Automatic Methane Potential Test System) in triplicate. Anaerobic digestion was performed in 500 mL sealed batch flasks using a 400 mL working volume at 37 °C for 30 days until no gas was detected with a 1:1 based on VS mixture ratio of substrates and inoculum (7.66 g of total VS) [50].

The inoculum used in this experiment was obtained from the Datansha Sewage treatment plant in Guangzhou, China, and cultivated in a mesophilic anaerobic fermentation tank in our laboratory over a long period, which has a characteristic of total solids (TS) 6.74%, volatile solids (VS) 1.00%, and pH 7.3–7.5. Before biomethane production, the inoculum was pre-incubated at 37 °C for 1 week in a starving condition aiming at the reduction of endogenous biomethane production. Then N_2_ was purged into the bottles for 3–5 min to guarantee anaerobic conditions. Bottles containing pure inoculum as blank samples as well as microcrystalline cellulose and inoculum as control samples were simultaneously determined.

During biomethane production, samples were withdrawn for determining the value of TS, VS, COD, and ammonium nitrogen (NH_3_-N) based on the previously reported method [55, 56], and pH values were detected by a pH meter (Five Easy Plus, Mettler-Toledo, Australia).

### Statistical analysis

Analysis of standard errors and variance use SPSS version 16.0. Significance was analyzed and used when the *p* value < 0.05.

## Data Availability

All data generated during this study are included in this published article and its additional file.

## Abbreviations

SCB: sugarcane bagasse
DER: *Dioscorea composita* Hemls. extracted residue
SSF: simultaneous saccharification and fermentation
COD: chemical oxygen demand
HPLC: high-performance liquid chromatography
RID: Refractive Index Detector
BMP: biological methane potential
AMPTS: Automatic Methane Potential Test System
TS: total solid
VS: volatile solid
AIL: acid-insoluble lignin
ASL: acid-soluble lignin

## Footnotes

1 Biofuels only accounted for less than 2% of the world’s liquids demand in 2017 [1].

2 Most climate model scenarios rely on carbon dioxide removal (CDR) technologies to limit future temperature rises [6, 7].
